# Expression of Prostate-specific Membrane Antigen (PSMA) in Papillary Renal Cell Carcinoma - Overview and Report on a Large Multicenter Cohort

**DOI:** 10.7150/jca.63509

**Published:** 2022-03-14

**Authors:** Stefanie Zschäbitz, Franziska Erlmeier, Christine Stöhr, Edwin Herrmann, Iris Polifka, Abbas Agaimy, Lutz Trojan, Philipp Ströbel, Frank Becker, Christian Wülfing, Peter Barth, Michael Stöckle, Michael Staehler, Christian Stief, Axel Haferkamp, Markus Hohenfellner, Stephan Macher-Göppinger, Bernd Wullich, Joachim Noldus, Walburgis Brenner, Frederik C. Roos, Bernhard Walter, Wolfgang Otto, Maximilian Burger, Andres Jan Schrader, Yvonne Mondorf, Arndt Hartmann, Philipp Ivanyi, Sandra Steffens

**Affiliations:** 1 Dept. of Medical Oncology, National Center of Tumor Diseases, University Hospital Heidelberg, 69120 Heidelberg, Germany; 2 Institute of Pathology, University Hospital Erlangen-Nuernberg, Friedrich Alexander University (FAU), 91054 Erlangen, Germany; 3 Department of Urology, University Hospital Münster, 48149 Münster, Germany; 4 Department of Urology, University Hospital Göttingen, 37075 Göttingen, Germany; 5 Institute of Pathology, University Hospital Göttingen, 37075 Göttingen, Germany; 6 Department of Urology and Pediatric Urology, University Hospital Saarland (UKS), 66421 Homburg, Germany; 7 Department of Urology, University Hospital Marburg, 35037 Marburg, Germany; 8 Department of Urology, University Hospital Munich, 81337 Munich, Germany; 9 Department of Urology, University Hospital Heidelberg, 69120 Heidelberg, Germany; 10 Institute of Pathology, University Hospital Mainz, 55131 Mainz, Germany; 11 Department of Urology and Pediatric Urology, University Hospital Erlangen, 91058 Erlangen, Germany; 12 Department of Urology, Marien-Hospital Herne, Ruhr University Bochum, 44625 Herne, Germany; 13 Department of Urology, University Hospital Mainz, 55131 Mainz, Germany; 14 Department of Urology, University Hospital Frankfurt, 60590 Frankfurt/Main, Germany; 15 Department of Urology, University Hospital Regensburg, 93053 Regensburg, Germany; 16 Department of Hematology, Hemostasis, Oncology and Stem Cell Transplantation, Hannover Medical School, 30625 Hannover, Germany; 17 Present address: Institute of Urology, Prosper-Hospital GmbH, 45659 Recklinghausen, Germany; 18 Present address: Urological Group and Clinic Derouet/Pönicke/Becker, Boxberg Centre, 66538 Neunkirchen, Germany; 19 Present address: Department of Urology, Asklepios Clinics Altona, 22763 Hamburg, Germany; 20 Present address: Institute of Pathology/Gerhard-Domagk Institute, University Hospital Münster, 48149 Münster, Germany; 21 Present address: Department of Urology and Pediatric Urology, University Hospital Mainz, 55131 Mainz, Germany; 22 Present address: Department of Gynecology, University Hospital Mainz, 55131 Mainz, Germany; 23 Present address: Department of Urology, Kreiskliniken Altötting-Burghausen, 84489 Burghausen, Germany; 24 Present address: Department of Hematology, Hemostasis, Oncology and Stem Cell Transplantation, Hannover Medical School, Hannover, Germany

**Keywords:** prostate specific membrane antigen, papillary renal cell carcinoma, kidney cancer

## Abstract

Prostate specific membrane antigen (PSMA) is an emerging diagnostic and therapeutic target in prostate cancer. ^68^Ga-PSMA-labeled hybrid imaging is used for the detection of prostate primary tumors and metastases. Therapeutic applications such as Lutetium-177 PSMA radionuclide therapy or bispecific antibodies that target PSMA are currently under investigation within clinical trials. The expression of PSMA, however, is not specific to prostate-tissue. It has been described in the neovascular endothelium of different types of cancer such as breast cancer, and clear cell renal cell carcinoma (ccRCC). The aim of this study was to analyze PSMA expression in papillary RCC (pRCC) type 1 and type 2, the most common non-ccRCC subtypes, and to evaluate the potential of PSMA-targeted imaging and treatment in pRCC. Formalin-fixed, paraffin-embedded tissue samples of primary tumors were analyzed for PSMA expression by immunohistochemistry. Out of n=374 pRCC specimens from the multicenter PANZAR consortium, n=197 pRCC type 1 and n=110 type 2 specimens were eligible for analysis and correlated with clinical data. In pRCC type 1 PSMA staining was positive in 4 of 197 (2.0%) samples whereas none (0/110) of the pRCC type 2 samples were positive for PSMA in this large cohort of pRCC patients. No significant PSMA expression was detected in pRCC. Reflecting current clinical evaluation of PMSA expression in RCC do not encourage further analysis in papillary subtypes.

## Introduction

Papillary renal cell carcinoma (pRCC) is the second most common type of RCC according to the current (2016) classification of the World Health organization (WHO) and attributes to approximately 10-15% of RCC cases 1, 2. It is divided into two subtypes, type 1 and type 2. Type 2 tumors are associated with unfavorable clinical outcomes compared to type 1 pRCC 3-5. Comprehensive molecular characterization revealed that alterations in the MET*** (***mesenchymal-epithelial transition) gene are a common feature of type 1 pRCC whereas type 2 pRCC is a heterogenous disease with many subtypes. In pRCC type 2 mutations in SETD2, fumarat hydratase (FH) and CKDN2A silencing as well as TFE3 fusions have been described 6.

Prostate specific membrane antigen (PSMA; glutamate carboxypeptidase II) is a type 2 transmembrane glycoprotein that consists of three parts: an 18 amino acid intracellular part, an 24 amino acid transmembrane part, and an 707 amino acid external part 7. PSMA is located on the short arm of chromosome 11 and has *in vitro* neuropeptidase and folate hydrolase activity 8. Initially, PSMA was considered to be exclusively expressed in prostatic tissue. An upregulation of PSMA in prostate cancer specimens can be observed and there is growing evidence that androgen deprivation drives this increase of PSMA 9. The extent of immunohistochemical PSMA expression in prostate cancer tissue is correlated to tumor uptake on ^68^Ga-PSMA positron emission tomography (PET)/ computer tomography (CT) 10.^ 68^Ga-PSMA-labeled hybrid imaging is nowadays widely used in Europe and Australia to detect recurrent prostate cancer as it outperforms conventional imaging techniques 11, 12. Although not currently approved, PSMA directed radioligand therapies with Lutetium-177 (^177^Lu-PSMA-617; ^177^Lu-PSMA-I&T) or Actinium-225 (^225^Ac-PSMA-617) or others are under investigation within clinical trials and are also used off-label for treatment of refractory metastatic prostate cancer 13-19.

Despite its name PSMA is not specific to prostate tissue. Physiologic expression was described in salivary glands, proximal renal tubules, brain, and small intestine tissues 20, 21. PSMA expression has been found in the neovascular endothelium of several types of cancer, e.g. breast cancer, colorectal cancer, non-small cell lung carcinoma, and RCC 21-24. Among RCC samples, PSMA expression has been studied in larger cohorts of clear cell RCC (ccRCC). Only small sample sizes of non-ccRCC were included in those analyses and results were inconclusive. Within ccRCC PSMA expression was found to be increased in vena cava tumor thrombi compared to renal tumor mass suggesting a potential mechanism for progression and malignant neovascularization 25.

Small case series have reported promising results of PET/CT in RCC patients using different PSMA directed radiotracers potentially outperforming conventional imaging modalities 26-29. Clinical trials are underway exploring the use of ^68^Ga-PSMA-labeled hybrid imaging for patients with PSMA positive tumors other than prostate cancer (Table 1).

The aim of our investigation was to determine the expression of PSMA in pRCC type 1 and 2 and to evaluate PSMA as a potential diagnostic or therapeutic target in these RCC subtypes.

## Material and Methods

### Patient cohort

**Figure 1:**
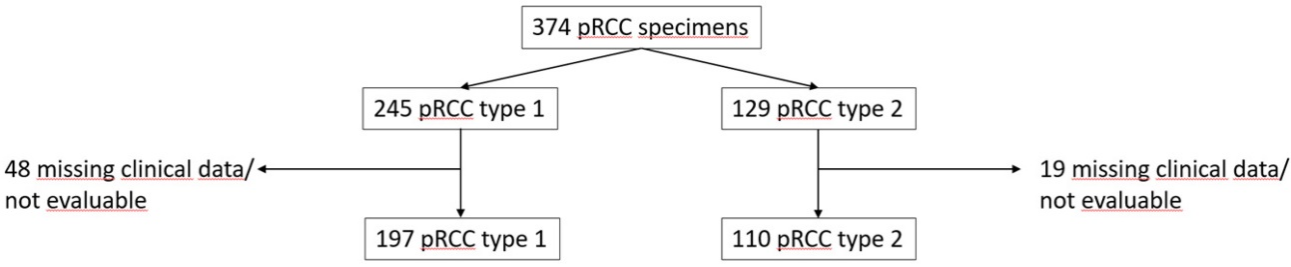
Consortium diagram of PANZAR cohort.

Routine kidney surgery due to kidney tumor was performed between 1994 and 2007. A total of 374 pRCC type 1 and 2 specimens, 245 (65.5%) type 1 and 129 (34.5%) type 2, from the multicentric PANZAR consortium were analyzed (Figure 1).

Primary tumor samples were perceived from the PANZAR contributing partners (in alphabetical order: Erlangen, Heidelberg, Herne, Homburg, Mainz, Mannheim, Marburg, Münster, LMU Munich, TU Munich and Regensburg). Detailed clinical data of the cohort have been previously published 31. For the analysis of PSMA expression samples of n=307 patients were available. Papillary subtype and pathological TNM staging were determined by an experienced uropathologist (AH). For each case, the papillary subtype was defined according to 2004 WHO tumor classification, and pathological TNM was performed according to the 2002 TNM classification. Grading was performed according to ISUP and WHO. Papillary type 1, or type 2 RCC were defined as previously reported 32. Patients and pathological data were accessed retrospectively. The study was performed according to the standards established in the Declaration of Helsinki and in concordance with each local ethic committee recommendations.

### Tissue preparation and Immunohistochemistry

One representative area of the pRCC tumors was selected to construct tissue microarrays. PSMA expression was determined by immunohistochemistry (IHC). 2 μm TMA slides were stained for PSMA (Anti-PSMA, clone 3E6, M3620, DAKO, dilution 1:20) with a fully automated Dako Autostainer (Dako, Agilent pathology systems). The antibody is established in routine pathological diagnostic. Antigen retrieval was accomplished at pH=7.2. The bound antibody was visualized with an HRP-conjugated secondary antibody (Dako) and the diaminobenzidine chromogen (Dako). Sections were briefly rinsed in tap water, counterstained with Mayer's Hematoxylin solution and then mounted. All stained tissue samples were assessed in a blind way by pathologists (FE, AH). The evaluation was performed under a Leitz ARISTOPLAN light microscope (Leica Microsystems, Germany) with a x10 eyepiece, a 22-mm field of view and x40 objective lens (Plan FLUOTAR x40/0.70). All microarrays were stained in the same batch, and positive and negative controls were included according to the antibody manufacturer's instructions. Prostate cancer tissue was used as positive control. The immunohistochemical results were reported as staining intensity and percentage of positively staining cells following the immunoreactive score. The staining reaction was classified as positive (>/=1%).

### Statistical Analyses

Descriptive statistics were performed with SPSS v25 (Armonk, NY, USA). Two-sided p-values below 0.005 were considered statistically significant.

## Results

Tissue microarrays of n=307 patients with pRCC type 1 (n=197 (64.2%)) and type 2 (n=110 (35.8%)) were eligible for analysis. Clinical and pathological parameters of the analyzed cohort are displayed in Table 2.

**Figure 2:**
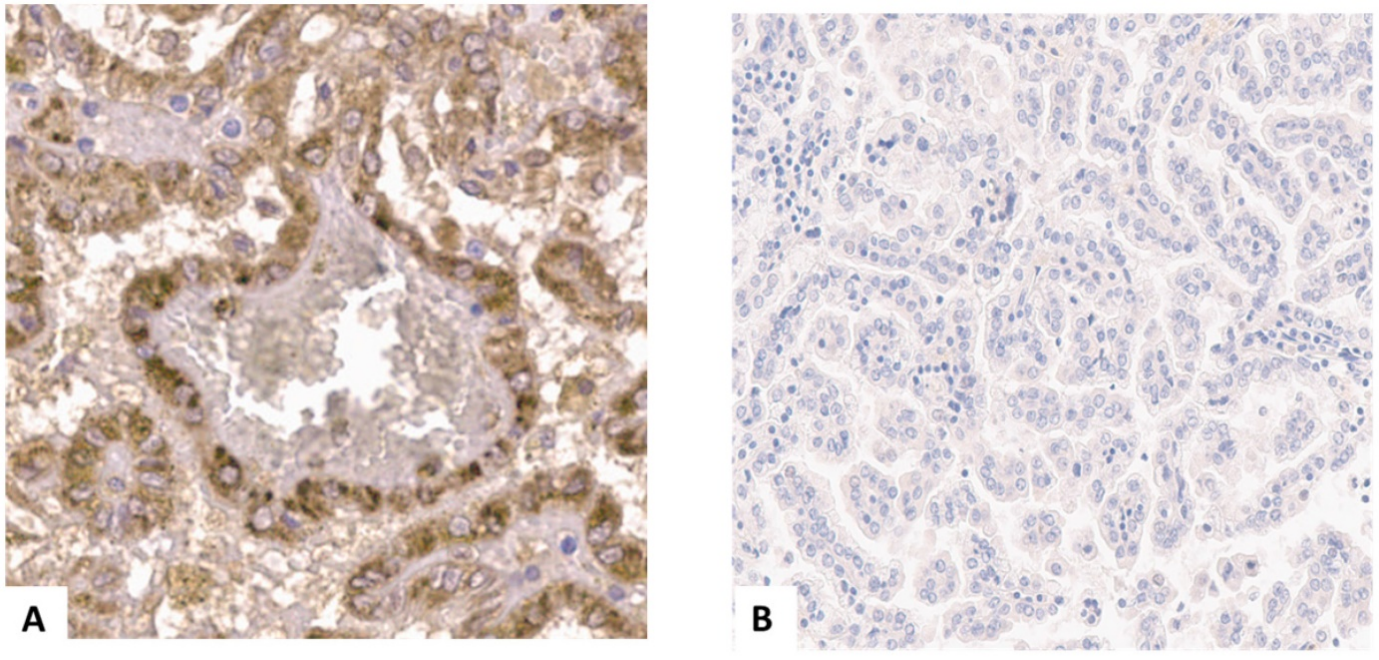
** (A)** Positive prostate specific membrane antigen (PSMA) staining in a papillary renal cell carcinoma type 1 specimen. **(B)** negative PSMA staining in a papillary renal cell carcinoma type 1 specimen.

In pRCC type 1 PSMA staining was positive in 4/197 (2.0%) of specimens, and in none (0.0%) of type 2 specimens (Figure 2A and 2B). In these four samples intensity of PSMA staining was strong in almost 100% of the area. PSMA staining was detected primarily in the cell membrane and cytoplasm of tumor cells. Furthermore, a weak staining reaction was detected in single endothelial cells of the neovasculature in these cases. Therefore, we used a binary cutoff system with positive or negative staining reaction.

In all four PSMA positive tumors stage was pT1 cN0 cM0 and grading was G2. No significant association between clinicopathological parameters nor follow up parameters and PSMA positivity were observed (Table 2, outcome parameters not shown).

## Discussion

PSMA expression by immunohistochemistry has been explored for many non-prostatic tumor types and among them RCC. Diagnostic, as well as therapeutic trials are ongoing in RCC addressing PSMA as target. In non-malignant renal tissue PSMA is found in the proximal renal tubules 20, 23. Upregulation of PSMA in the neovasculature of ccRCC samples has been described whereas the overall expression level in RCC is low 21, 22, 33. Since biology of ccRCC and non-ccRCC is different, data on PSMA expression in non-ccRCC, like pRCC is important to evaluate, to address the potential role of PSMA in this subtype of RCC.

Data on PSMA expression in pRCC has been reported, however reported sample sizes are considerably small, and results are inconclusive. Spatz et al. report on 257 RCC samples of all subtypes analyzing PSMA expression in the vasculature of renal primary tumors, wherein 22 (8.6%) papillary subtypes were included 24. Herein, in particular PSMA expression was found in 82% of the ccRCC specimen and only in 13.6% of the pRCC specimen (n=3; weakly positive (n=2), strongly positive (n=1)). An association with PSMA expression and clinicopathological risk parameters was primarily found for ccRCC only, not in non-ccRCC. Al-Ahamdie et al. investigated 75 nephrectomy specimens, among them 15 pRCC samples 22. Whereas ccRCC specimen showed a diffuse (24/30, 80%) and mostly a strong (25/30, 83%) PSMA staining pattern, in pRCC specimen no diffuse PMSA expression and only focal expression (11/15, 73%) with minimal strong expression (5/15, 33%) was identified. Among all RCC subtypes analyzed ccRCC showed the most, non-ccRCC lesser and pRCC the least intense and extensive PSMA expression. No association of clinicopathological parameters and PSMA expression was found in pRCC, neither in type 1 nor 2. Kinoshita et al. analyzed 18 renal tumor samples. While 11 samples were PSMA negative, they found a moderate staining intensity in 2 of 2 pRCC and 2 of 9 ccRCC samples and a weak PSMA staining in 3 of 9 ccRCC samples 21. Baccala et al found that positive PSMA staining was detectable in 76.2% of ccRCC, 31.2% of chromophobe RCC, 52.6% of oncocytoma, and 0% of PRCC samples 23. Evangelista et al give an overview on PSMA PET imaging in RCC 34 that has been used in small case series or proof-of-concept trials. While for ccRCC results indicate that further usage should be explored no statement can be made regarding pRCC due to small sample size.

PANZAR is the largest pRCC cohort reported to date. To elucidate whether PSMA might play a role in pRCC, due to the heterogenous data about this histological subset we analyzed PSMA expression on tissue microarrays of 197 patients with pRCC type 1 and 110 patients with pRCC type 2. Our analysis showed a rare expression in pRCC type 1 and no PSMA expression in pRCC type 2 samples. Accordingly, no association with clinicopathological parameter could be observed. Our results are not consistent with all previously published data. However, in concordance to prior reports e.g. 23, we could strongly underline a limited impact of PSMA expression in pRCC. Differences in amount of positivity among the different analyses remain elusive, but limitations of all analyzes were the same: methodology of IHC, different ages of specimen cohorts, as well as their retrospective character. A further limitation is the use of tissue microarrays, because only a small part of tumor can be analyzed. This small section often shows only several neovasculature vessels, which leads to a limited significance. However, the tissue micro-arrays (TMAs) of the PANZAR cohort have successfully been used in several former analyses and we consider those samples as representative for pRCC. Also, thickness of slides of the TMA used in our analysis is within standard range (2-7 µm). Analysis of several samples per tumor specimen would have been possible. Considering missing clinical relevance in the case of heterogenous expression (i.e. missing target for application of theranostic/ therapeutic approaches) we decided against further analyses.

Furthermore, PSMA is an unspecific antigen. It is expressed in prostate tissue and, to a lesser extent, in peripheral and central nervous system, small intestinal and salivary gland tissues. Cytoplasmatic and, to a lesser degree, membranous PSMA expression has been recently documented in 11% of analyzed urinary bladder adenocarcinomas 35. The lack of specificity could explain the positive staining reaction in several cases.

What to consider from our findings from bench side to bed side? Case series on ^68^Ga-PSMA targeted PET imaging in RCC patients showed encouraging results for ccRCC in detecting metastatic lesions that were not revealed in conventional imaging (30, 36; Reviews: 37, 38). In pRCC patients, however, ^68^GA- PSMA-PET-PET CT did not detect additional metastases compared to conventional imaging modalities 39. SUV_max_ in pRCC was also reported to be lower than in other RCC types. As sample sizes were small (number of pRCC patients between 1-3 patients) results were discussed not to be representative 39. The missing expression of PSMA as reported for the PANZAR cohort, however, explains that finding well. Larger trials to investigate PSMA hybrid imaging in RCC should probably exclude pRCC.

Therapeutic applications targeting PSMA have found entrance into the treatment of prostate cancer and are currently investigated for other cancer entities 40. Besides Lutetium-177-PSMA or Actinium-225-PSMA radionuclide therapy bispecific antibodies that target PSMA have been investigated in prostate cancer 41. PSMA targeted CAR-T cell therapy might offer an additional treatment option for PSMA positive tumors and probably will enter clinic in the near future 42. However, as for imaging we do not consider PSMA directed therapeutics promising for patients suffering from pRCC.

## Conclusions

No relevant PSMA expression and consequently no relevant clinicopathological association with PMSA was detected in the pRCC PANZAR consortium cohort. Based on our data, a prognostic value of PMSA in pRCC is unlikely. Further on, due to missing PSMA expression in pRCC our findings point out that neither in pRCC type 1 nor type 2 PSMA targeted PET imaging or PMSA targeted therapeutical approaches seem to be reasonable. Whether PSMA targeted PET imaging and treatment modalities might expand the diagnostic and therapeutic landscape in other RCC subtypes needs to be further elucidated.

## Figures and Tables

**Table 1:** Overview of prospective clinical trials (diagnostic) targeting Prostate Specific Membrane Antigen (PSMA) in Renal Cell Carcinoma.

NCT identifier	Tumor entity	Tracer, mode of imaging	Trial phase	Status
NCT03427476	Metastatic RCC	^18^F-CTT1057 PET/CT or MRT	1	Completed
NCT02687139	Clear cell RCC	^18^F-DCFPyL PET/CT	1	Completed, (30)
NCT03387514	Metastatic clear cell RCC	^18^F-DCFPyL PET/CT	2	Recruiting
NCT03073395	Metastatic RCC	^68^Ga-P16-093 PET/CT	1	Recruiting
NCT02978586	breast cancer, lung cancer, and other tumor types know to express “PSMA”	^68^Ga-PSMA PET/CT	-	Recruiting
NCT03453528	Advanced/metastatic solid tumors	^68^Ga-PSMA PET/CT	-	Recruiting
NCT03073395	Metastatic RCC	^68^Ga-P16-093 PET/CT	1	Recruiting
NCT03841760	PSMA-expressing non-prostate tumor	^18^F-DCFPyL PET/CT or ^68^Ga-PSMA-11 PET/CT	2	Recruiting
NCT04147494	RCC, solid cancer	^68^Ga-FAPI-46 PET/CT or ^68^Ga-PSMA PET/CT	1	Recruiting

**Table 2:** Clinical and pathological parameters of the analyzed cohort.

Variable	pRCC Type 1 PSMA^-^ n=193 (98.0%)	pRCC Type 1 PSMA^+^ n= 4 (2.0%)	p-value	pRCC Type 2 PSMA^-^ n=110 (100.0%)	pRCC Type 2 PSMA^+^ n=0 (0%)	p-value
Age [years] median (range)	63.0 (15-86)	66.5 (57-77)	p=.400	66.0 (18-85)		
NE (n)	34	0		26		
Sex			p=.816			
Male, n (%)	126 (65.3)	3 (75.0)		64 (58.2)		
Female, n (%)	32 (16.6)	1 (25.0)		20 (18.2)		
NE, n (%)	35 (18.1)	0 (0.0)		26 (23.6)		
TNM Stage			p=.382			
pT1, n (%)	106 (54.9)	2 (50.0)		37 (33.6)		
pT2, n (%)	35 (18.1)	2 (50.0)		14 (12.7)		
pT3, n (%)	17 (8.8)	0 (0.0)		30 (27.3)		
pT4, n (%)	0 (0.0)	0 (0.0)		1 (0.9)		
NE, n (%)	35 (18.1)	0 (0.0)		28 (25.5)		
Grade			p=.367			
G1, n (%)	45 (23.3)	0 (0.0)		9 (8.2)		
G2, n (%)	107 (55.4)	4 (100.0)		52 (47.3)		
G3, n (%)	4 (2.1)	0 (0.0)		19 (17.3)		
G4, n (%)	0 (0.0)	0 (0.0)		0 (0.0)		
NE, n (%)	37 (19.2)	0 (0.0)		30 (27.3)		
Lymph node metastasis^ #^			p=.751			
N-, n (%)	187 (94.9)	4 (100.0)		93 (84.5)		
N+, n (%)	6 (3.1)	0 (0.0)		17 (15.5)		
NE, n (%)	0 (0.0)	0 (0.0)		0 (0.0)		
Distant metastasis^#^			p=.819			
M-, n (%)	153 (79.3)	4 (100.0)		65 (59.1)		
M+, n (%)	2 (1.0)	0 (0.0)		14 (12.7)		
NE, n (%)	38 (19.7)	0 (0.0)		31 (28.2)		
Locally Advanced disease			p=.471			
T1/T2 N0M0, n (%)	138 (71.5)	4 (100.0)		48 (43.6)		
T3/4 and/or N+ and/or M+, n (%)	18 (9.3)	0 (0.0)		31 (28.2)		
NE, n (%)	37 (19.2)	0 (0.0)		31 (28.2)		

^#^ at time of renal surgery. Legend: N- = lymph node status unknown or tumor cells absent from regional lymph nodes, N+ = regional lymph node metastasis present, NE = not evaluable.

## Abbreviations

ccRCC: clear cell renal cell carcinoma
CT: computer tomography
PET: positron emission tomography
pRCC: papillary renal cell carcinoma
PSMA: prostate specific membrane antigen
RCC: renal cell carcinoma
SUV: standardized uptake value
